# Synergistic Effects of Heat-Killed Kefir Paraprobiotics and Flavonoid-Rich Prebiotics on Western Diet-Induced Obesity

**DOI:** 10.3390/nu12082465

**Published:** 2020-08-16

**Authors:** Kun-Ho Seo, Jaewoon Jeong, Hyunsook Kim

**Affiliations:** 1 Center for One Health, College of Veterinary Medicine, Konkuk University, Hwayang-dong, Gwangjin-gu, Seoul 05029, Korea; bracstu3@konkuk.ac.kr (K.-H.S.); jjeong@upei.ca (J.J.); 2 Department of Health Management, Atlantic Veterinary College, University of Prince Edward Island, Charlottetown, PE C1A 4P3, Canada; 3 Department of Food and Nutrition, Hanyang University, 222 Wangsimni-ro, Seongdong-gu, Seoul 04763, Korea

**Keywords:** heat-killed lactic acid bacteria, paraprobiotics, obesity, prebiotics, wine grape seed flour

## Abstract

The synergistic anti-obesity effect of paraprobiotic heat-killed lactic acid bacteria (HLAB) and prebiotics has not been studied. To determine the anti-obesity properties of prebiotic polyphenol-rich wine grape seed flour (GSF) and paraprobiotic HLAB, C57BL/6J mice were administered a high-fat and high-fructose diet (HFFrD) with 5% microcrystalline cellulose (CON), HFFrD supplemented with 2.5% GSF, HFFrD with orally administered HLAB, or HFFrD with a combination of GSF and orally administered HLAB (GSF+HLAB) for 8 weeks. Compared with the CON group, the GSF and HLAB groups both showed significant reductions in HFFrD-induced body weight gain and adipose tissue weights (*p* < 0.05). Interestingly, combined supplementation with GSF and HLAB revealed statistically significant synergistic effects on body weight gain, visceral adiposity, and plasma triacylglycerol concentrations (*p* < 0.05). The synergistic action was significantly related to a decreased adipocyte gene expression in fatty acid synthesis and inflammation metabolism. In conclusion, the combination of prebiotic GSF and paraprobiotic kefir HLAB is potentially useful, as natural food ingredients, in the prevention of obesity and obesity-related diseases, especially for immunocompromised individuals.

## 1. Introduction

Obesity is a serious risk factor for several chronic diseases, such as hypertension, cardiovascular disease (CVD), type 2 diabetes, respiratory disorders, and cancers. It incurs tremendous medical costs in both developed and developing countries [1]. Therapeutic drugs to treat obesity reportedly cause several side effects related to blood pressure, hepatic failure, pancreatitis, and headaches [2]. Therefore, safe and effective natural products for the treatment of obesity are desperately needed.

Consumers’ interest in consuming fermented foods containing live micro-organisms (probiotics) for their health benefits has driven research and marketing [3]. The potential anti-obesity effects of probiotics have been reported in 3T3-L1 preadipocytes. In addition, *Lactobacillus plantarum* FH185, *L. plantarum* K21, *L. gasseri* BNR17, *L. gasseri* SBT2055, and *Bifidobacterium breve* B-3 were found to prevent diet-induced body weight gain and obesity-related metabolic disturbances in rats and mice [4].

Most studies on the health benefits of foods containing probiotic lactic acid bacteria have focused on live bacteria and, in fact, the live bacteria content is often related to efficacy. More recently, foods containing nonviable microorganisms or paraprobiotics that confer health benefits have gained research interest. Paraprobiotics are referred to as “non-viable microbial cells (intact or broken) or crude cell extracts (i.e., with complex chemical composition), which, when administered (orally or topically) in adequate amounts, confer a benefit on the human or animal consumer” [5]. Recent studies have shown that the health benefits of a diet containing heat-killed lactic acid bacteria (HLAB) include reduced body weight gain, immunomodulation, increased intestinal barrier integrity, and a decreased number of enteropathogens [4,6]. Moreover, they have advantages over live probiotics with regard to safety for immunocompromised subjects and the ease of standardization and preparation [4].

Kefir, a fermented dairy beverage that originated in Eastern Europe, is a natural probiotic complex. It includes probiotic lactic acid bacteria (*Lactobacillus*, *Lactococcus*, *Leuconostoc*, and *Streptococcus* spp.), acetic acid bacteria (*Acetobacter*), and yeasts (*Saccharomyces*, *Kluyveromyces*, *Torula*, and *Candida* spp.) in a symbiotic relationship. We and others have shown several health benefits of consuming kefir, which include cholesterol-lowering, antimicrobial, immuno-modulatory, anti-inflammatory, and anti-obesity effects [7,8,9]. In addition, we have recently revealed the probiotic effect of *Lactobacillus kefiri* DH5 (LKDH5), a bacterium isolated from kefir fermented milk—it inhibits the development of obesity and non-alcoholic liver diseases [10].

Our previous studies showed that the supplementation of diet with wine grape seed flour (GSF), a byproduct of winemaking, suppressed high-fat (HF) diet-induced body weight gain, hepatic steatosis, visceral adiposity, and hyperlipidemia by the modulation of genes involved in oxidative stress, inflammation, and cholesterol and fatty acid metabolism [11,12,13]. These effects were associated with a high content of flavonoids in GSF [13]. We have recently shown that the combination of flavonoid-rich grape seed flour (GSF) and live kefir-derived lactic acid bacteria (LAB) improved HF-induced obesity and obesity-related metabolic disorders in a synergistic manner [14]. However, the combined action of prebiotic GSF and paraprobiotic kefir HLAB has not been evaluated.

Our aim was to investigate the effect of kefir HLAB in combination with GSF, a bioactive polyphenol source, for their individual and symbiotic benefits in high-fat and high-fructose diet (HFFrD)-fed obese mice.

## 2. Materials and Methods

### 2.1. Animals and Diets

Four-week-old male C57BL/6J mice purchased from Orient Bio, Inc. (Sungnam, Korea) were housed under a 12 h/12 h light/dark cycle in an environmentally controlled room (temperature, 20–22 °C; relative humidity, 60%). The study was performed in accordance with all institutional and national guidelines for animal care and approved by the Animal Experiment Ethics Committee at Konkuk University (study protocol #KU16046 and KU17020). Prior to experimental diet initiation, the mice were acclimated for 1 week, during which mouse chow (PMI International, Redwood, CA, USA) and water were made available ad libitum. Thereafter, the mice (*n* = 10 per each group) were fed a obesogenic, high-fat/high-fructose diet (HFFrD) containing 5% microcrystalline cellulose (CON, control diet; Dyets Inc, Bethlehem, PA), 2.5% grape seed flour (GSF), 2.5% GSF+HLAB, or HLAB for 8 weeks ad libitum (Table 1). Ninety-nine percent of C57BL/6J mice became obese after eight weeks of the HFFrD diet (mice fed HFFrD diet increased body weight gain by 57% compared to chow diet group). HLAB suspended in saline were administered orally, and the CON and GSF groups were also administered oral saline. The composition of macronutrients and total dietary fiber, containing approximately 17%, 36%, and 47% calories from protein, carbohydrates, and fat, respectively, was matched across the diets (Table 1). Chardonnay GSF was purchased from AprèsVin Enterprise, Inc. (Prosser, WA, USA), and its total phenolic content was analyzed using the Folin–Ciocalteu method. The HLAB were prepared from *Leuconostoc mesenteroides* 4 (LMDH4) and *Lactobacillus kefiri* DH5 (LKDH5) previously isolated from kefir grain and cultured in lactobacilli Man, Rogosa, and Sharpe (MRS) broth (Difco, BD Biosciences, USA) at 37 °C for 48 h. After mass culture, the LAB were heat killed at 70 °C for 30 min. A 1 × 10^10^ CFU/mL culture of *L. mesenteroides* 4 (LMDH04) and 1 × 10^9^ CFU/mL of *L. kefiri* DH5 (LKDH5) were lyophilized. Before administration to the animals, the HLAB were rehydrated with sterilized distilled water at room temperature for 0.5–1 h. The HLAB were orally administered at 10 mL/kg body weight. The saline groups were administered by gavage at 10 mL of saline/kg body weight. Body weights were measured weekly, and food intake was monitored twice every week.

### 2.2. Sample Collection and Plasma Lipid Analysis

Following a 12-h fasting period, the mice were anesthetized in an induction chamber, using a vaporizer (VetEquip, Livermore, CA) containing 4% isoflurane (Phoenix Pharmaceutical, St. Joseph, MO, USA) with an oxygen flow of 1 L/min. Blood samples were collected using the cardiac puncture method using syringes previously applied with potassium EDTA solution (15% *w*/*v*). The plasma was collected and centrifuged at 4500 rpm for 10 min. After weighing, epididymal adipose tissue was immediately frozen in liquid nitrogen for further analysis.

Plasma lipoprotein cholesterol, triglycerides, and glucose concentrations were measured using a biochemical automatic analyzer (HITACHI, Tokyo, Japan).

### 2.3. Real-Time RT-PCR

Total RNA from epididymal adipose tissue was obtained using a TRIzol Plus RNA Purification Kit (Invitrogen, Life Technologies, Carlsbad, CA, USA). The cDNA was synthesized with 2 μL of total RNA using a PrimeScript RT Reagent Kit (Takara Biotechnology, Japan). A 2 μL sample of the diluted cDNA (1:10) was mixed with SYBR Green Supermix (Bio-Rad Laboratories, Hercules, CA, USA) as described in our previous study [14]. The sequences of the primers are presented in Table 2. Primers were verified by size and sequence analysis of the PCR products. Differences in mRNA expression were determined after normalization to the expression of 36B4 mRNA using the ^ΔΔ^CT method.

### 2.4. Isolation of Cellular Exopolysaccharide (EPS) from Kefir Lactic Acid Bacteria

Cellular EPS from kefir LAB was extracted according to the previous method [19] with slight modifications. *L. kefiri* (LKDH1, 3, and 5) and *L. mesenteroides* (LMDH04, 6, 7, 8, and 9) isolated from kefir grain (Konkuk University, Korea) [10] were used. LAB strains were cultured in MRS broth for 48 h. The cultured broth was incubated with 80% trichloroacetic acid (Sigma-Aldrich, St. Louis, MO, USA) for 30 min in a shaking incubator. The mixture was centrifuged at 8000× *g* for 20 min at 40 °C. Two volumes of ethanol were added to the supernatant and kept overnight at 40 °C. The precipitate was washed by suspending with two volumes of ethanol. The ethanol precipitation wash was repeated twice. The final precipitate was suspended in deionized water and dialyzed in deionized water for 48 h. The product was then lyophilized. Protein content in the EPS was analyzed using the Bradford method [20]. The lyophilized EPS had less than 0.01% of protein in dry matter.

### 2.5. 3T3-L1 Cell Culture

The 3T3-L1 preadipocyte cells were obtained from the American Type Culture Collection (ATCC, Manassas, VA, USA). Cells were grown in Dulbecco’s modified Eagle’s medium (DMEM) with high glucose (Gibco, Grand Island, NY, USA), 10% bovine serum (BS; Gibco), and 1% penicillin streptomycin (P/S; Gibco). Two days after confluence (day 0), cells were cultured with differentiation medium (DMEM supplemented with 10% fetal bovine serum (FBS; Gibco), 1% penicillin streptomycin (P/S), 0.5 mM 3-isobutyl-1-methylxanthine, 1 μM dexamethasone, and 10 μg/mL of insulin (Sigma-Aldrich, St. Louis, MO, USA)). After 48 h, culture medium was replaced with complete medium (DMEM containing 10% FBS, 1% P/S, and 10 μg/mL of insulin) and incubated for another 48 h. On day 4, the cells were cultured with DMEM containing 10% FBS and 1% P/S for another 24 h. During cell culture, cells were incubated in a CO_2_ incubator (Thermo Fisher Scientific Korea, Seoul, Korea) at 37 °C with 5% CO_2_.

After differentiation, the cells were stained with Oil red O followed by the addition of 100% isopropanol to elute the Oil red O dye. Extracted Oil red O was centrifuged at 10,000× *g* for 2 min and the supernatant was transferred to a 96-well plate. The absorbance (OD) was measured at 480 nm and the percentage of intracellular lipid accumulation was calculated as (OD_sample_/OD_control_) × 100.

### 2.6. Statistical Analysis

All data are shown as mean ± SEM. To determine the statistical significance of any difference between the control and treated groups, Duncan’s multiple range test of one-way variance (ANOVA) was performed. Pearson correlation analysis was applied to determine the correlation between mRNA gene expression and metabolic obesity physiological biomarkers. All statistical analyses were performed using SPSS software v20.0 (IBM SPSS Statistics, Chicago, IL, USA).

## 3. Results

### 3.1. Effects of Wine GSF and HLAB Supplementation on Body Weight, Organ Weight, and Metabolic Parameters

**Figure 1:**
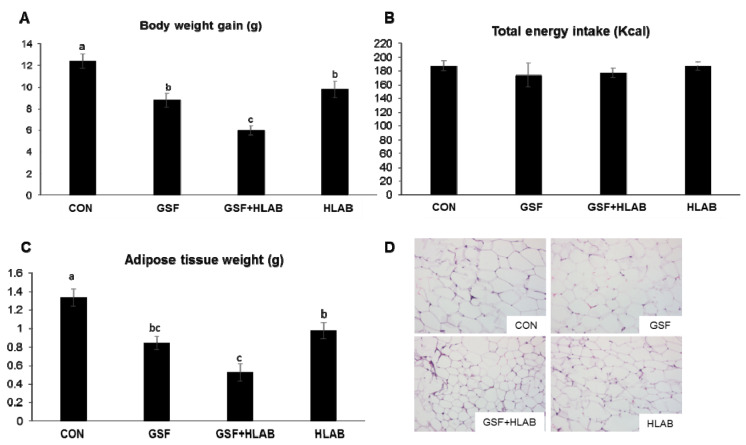
Effect of 2.5% wine grape seed flour (GSF) and heat-killed kefir lactic acid bacteria (HLAB) on C57BL/6J mice fed a high-fat and high-fructose diet (HFFrD) containing 5% microcrystalline cellulose (CON), 2.5% wine grape seed flour (GSF), 2.5% GSF with orally administered heat-killed kefir lactic acid bacteria (GSF+HLAB), and orally administered heat-killed kefir LAB (HLAB) for 8 weeks. (**A**) Body weight gain, (**B**) total energy intake, (**C**) adipose tissue weight, (**D**) Hematoxylin and Eosin (H&E) staining of adipose tissue. Data are expressed as mean ± SE; *n* = 8–10/group. Values not sharing a common letter differed significantly at *p* < 0.05.

The supplementation of the HFFrD with GSF, GSF+HLAB, and HLAB for 8 weeks significantly (*p* < 0.05) lowered body weight gain by 29%, 52%, and 21%, respectively, compared with that in the CON group, although total energy intake was not changed (Figure 1A,B). Compared with the CON group, the adipose tissue weights of the GSF, GSF+HLAB, and HLAB groups were significantly lowered by 37%, 61%, and 36%, respectively (*p* < 0.05) (Figure 1C). The liver weights were significantly lowered only by the GSF diet compared to the CON group (CON, 1.01 g ± 0.03; GSF, 0.86 ± 0.07; GSF+HLAB, 0.95 ± 0.02; LAB, 0.94 ± 0.04). Supplementation by GSF, GSF+HLAB, or HLAB did not affect plasma total cholesterol (T-C), HDL cholesterol, LDL cholesterol, or glucose concentrations in comparison with the CON group (Table 3). The HFFrD diet supplemented with GSF+HLAB significantly reduced plasma triglyceride concentration by 27% compared with the CON group (*p* < 0.05) (Table 3).

### 3.2. Analysis of Gene Expression in Adipose Tissue

**Figure 2:**
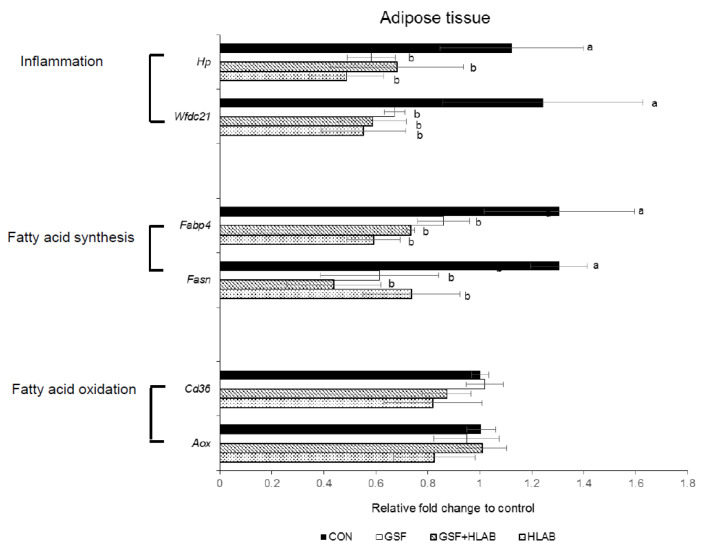
Effect of 2.5% wine grape seed flour (GSF) and heat-killed kefir lactic acid bacteria (HLAB) on gene expression levels in the adipose tissues of mice fed a high-fat and high-fructose diet (HFFrD) containing 5% microcrystalline cellulose (CON), 2.5% wine grape seed flour (GSF), 2.5% GSF with orally administered heat-killed kefir lactic acid bacteria (GSF+HLAB), and orally administered heat-killed kefir LAB (HLAB) for 8 weeks. Data are expressed as mean ± SE; *n* = 6/group. Values not sharing a common letter differed significantly at *p* < 0.05.

Haptoglobin (*HP*) gene expression was significantly downregulated by 48, 39, and 57%, respectively, in GSF, GSF+HLAB, and HLAB groups compared with the CON group (Figure 2). *Wfdc21*, coding for protein Wfdc21, expression was significantly lower by 46, 53, and 56% in GSF, GSF+HLAB, and HLAB, respectively, than the CON group (Figure 2). The fatty acid-binding protein 4 (*Fabp4*) gene in adipose tissue was significantly downregulated by 34, 43, and 55% after GSF, GSF+HLAB, and HLAB intake compared with the CON group (Figure 2). Compared with the CON group, the expression of the fatty acid synthase (*Fsan*) gene in adipose tissue in the GSF, GSF+HLAB, and HLAB groups was significantly downregulated by 52, 66, and 43%, respectively (Figure 2). The expressions of the cluster of differentiation 36 (*Cd36*) and Acyl-CoA oxidase (*Aox*) genes were not significantly different between the different diets (Figure 2).

### 3.3. Analysis of the Correlation between the Expression of Adipose mRNA and Metabolic Obesity Physiological Biomarkers

To determine whether the expression of adipose mRNA was associated with changes in metabolic obesity physiological biomarkers, correlation analyses between the adipose mRNA expression and three physiological biomarkers (body weight gain, adipose tissue weight, and plasma triglyceride concentration) were performed (Table 3). The expression of *Fabp4*, *Fasn*, *Wfdc21*, and *Hp* genes showed a significant (*p* < 0.05) positive correlation with body weight gain and adipose tissue weight (Table 4).

### 3.4. Inhibitory Activities of Cellular EPS of Kefir HLAB on Lipid Accumulation in 3T3-L1 Cells

**Figure 3:**
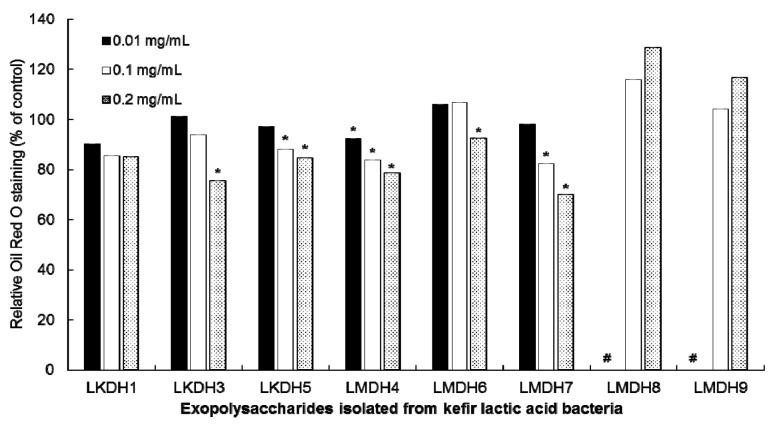
Inhibitory effects of cellular exopolysaccharide (EPS)s of heat-killed kefir lactic acid bacteria with increasing concentrations (0.01, 0.1, 0.2 mg/mL) on relative Oil red O staining content in 3T3-L1 adipocytes after differentiation (day 6). The cells differentiated in the presence of the heat-killed kefir LAB (HLAB) for 6 days after confluence. Values of the intracellular Oil red O staining of the treated cells are presented as percentages relative to the values of the untreated control. Data are expressed as mean ± SEM, * indicates a significant difference (*p* < 0.05) compared to control, # data were not measured.

To explore the bioactive component in HLAB against obesity, the inhibitory activities of cellular exopolysaccharides (EPSs) isolated from kefir *L. kefiri* (LKDH1, 3, and 5) and *L. mesenteroides* (LMDH4, 6, 7, and 8) on lipid accumulation were determined in 3T3-L1 cells using an Oil red O staining. Compared to the control, EPSs from LKDH5, LMDH4, and LMDH7 exhibited the greatest inhibition of lipid accumulation in a dose-dependent manner (Figure 3). All viabilities of 3T3-L1 cells treated with 0.01, 0.1, and 0.2 mg/mL of kefir LAB EPSs were greater than 90% compared to those of the control cells (data not shown).

## 4. Discussion

We have previously reported that a synbiotic combination of prebiotic polyphenol-rich grape seed flour (GSF) and live kefir-derived LAB improved HF-induced obesity-related metabolic disorders [14,21]. In the present study, we revealed that supplementation with paraprobiotic kefir HLAB resulted in a significant reduction of body weight gain and adipose tissue weight in HFFrD-induced obese mice. Furthermore, the combination of prebiotic GSF and paraprobiotic HLAB induced a synergistic, positive effect on obesity and hypertriglycemia.

Selected probiotic strains, such as *Lactobacillus* spp., *Bifidobacterium* spp., *Roseburia intestinalis*, and *Akkermansia muciniphila*, have shown positive effects in obesity-induced metabolic disorders in animals. However, their effects in humans are inconsistent [22,23,24]. Recently, several studies have revealed the health benefits of consuming HLAB on body weight, immunomodulation, intestinal barrier integrity, and enteropathogens [4,6]. HLAB and their cellular components exert anti-inflammatory and immunomodulating activities through toll-like receptor 4 (TLR4) signaling pathways in HepG2 cells [25] and THP-1 cells [26]. TLR4 is one of the pattern recognition receptors responding to invading pathogens and regulating innate and adaptive immune, as well as inflammatory, responses in liver and adipose tissues [27]. Mice lacking TLR4 have a significantly downregulated expression of genes targeted by peroxisome proliferator-activated receptors (PPARs). These studies suggest that HLAB modulate adipogenesis by regulating the expression of PPARs through TLR signaling. Consistent with previous studies, we show that kefir HLAB significantly reduced high fat- and fructose-induced body weight gain and adipose tissue weight gain compared with those in controls. More interestingly, HLAB significantly reduced the expression of genes associated with fatty acid synthesis (*Fabp4* and *Fasn*, PPAR γ target genes) and inflammation (*Hp* and *Wfdc21*), which indicates their possible role in fatty acid synthesis and anti-inflammation in adipose tissues. These beneficial effects were synergistically profound after HLAB were combined with 2.5% GSF. There were significant correlations between body weight gain, adipose tissue weight, and adipocyte expression of *Fabp4*, *Fasn*, *Wfdc21*, and *Hp*, indicating that the observed anti-obesity effect is mediated via the modulation of fatty acid synthesis and inflammation in adipose tissues.

We have shown that supplementation with 5% or 10% GSF inhibited HF diet-induced body weight gain, visceral adiposity, and hyperlipidemia by modulating the expression of genes related to oxidative stress, inflammation, cholesterol, and fatty acid metabolism in adipose tissues [11,12,13]. Combining 10% GSF and live LAB improved obesity and obesity-related metabolic biomarkers through the action of prebiotic GSF and probiotic LAB [14]. However, the observed synergistic effects were less significant due to the profound effect of 10% GSF itself [14]. The present study has demonstrated a strong synergistic anti-obesity action using 2.5% GSF and HLAB.

The GSF ingredient contains 5.78 g of protein, 8.66 g of fat, 17.3 g of carbohydrates, and 57.3 g of total dietary fiber in 100 g (Table 1). The composition of macronutrients and total dietary fiber, containing approximately 17%, 36%, and 47% calories from protein, carbohydrates, and fat, respectively, was matched across the diets (Table 1). When a diet contains low carbohydrate content, energy expenditure increases, resulting in weight loss [28]. Thus, the observed anti-obesity effect with HLAB is not related to the carbohydrate composition in the CON and GSF diets.

In our recent study [29], 5% EPS (called kefiran) extracted from kefir grain showed greater anti-obesity effect in high fat diet-induced obese mice compared to 5% β-glucan (BG) although it has less viscosity than BG. More interestingly, 5% EPS significantly increased the abundance of *Akkermansia* spp., a probiotic against metabolic diseases, compared to the control. Based on this study, it is hypothesized that the anti-obesity action of HLAB can be partially attributed to the cellular EPS component. Indeed, in the current study, inhibitory activity in the lipid accumulation in 3T3-L1 cells was greater in both EPSs from the HLAB, LKDH5 and LMDH4, that were used as paraprobiotics for the animal study. The amount of orally ingested kefiran in the previous mouse study was at least 500 times greater than in this study of the paraprobiotic HLAB. We estimate this based on 150 mg kefiran intake calculated from 3 g diet/d containing 5% kefiran and compared it to the 300 μg HLAB administered in this study. Taken together, while kefiran EPS has an effect, its potency compared to other cellular components in the nonviable LAB is considerably lower.

HLAB contain various cellular components, such as exopolysaccharides (EPSs), peptidoglycan, lipoproteins, and lipopeptides [30]. Polysaccharide A (PSA) from *Bacteroides fragilis* and EPS from *Lactobacillus rhamnosus* GG induce TLR2-dependent responses in 3T3-L1 adipocytes and T cells [19,31]. An additional function of EPS may be that EPS is a fermentable source of bacterial metabolites produced by the action of intestinal microbiota [32]. Our previous study demonstrated that the consumption of 5% kefiran (EPS extracted from kefir grain) prevented high fat-induced obesity in an animal model [29], which was associated with the modulation of intestinal microbiota. *L. kefiri* DH5 (LKDH5) and *L. mesenteroides* 4 (LMDH04) used for present animal study showed the greatest inhibitory pancreatic lipase activity [33]. Tannin polyphenols enriched in GSF have an inhibitory pancreatic lipase activity [34]. The relative abundance of *A. muciniphila* was increased by pancreatic enzyme therapy in chronic pancreatitis subjects [35], supporting a close relationship between pancreatic lipase activities and the modulation of intestinal microbiota. Pancreatic lipase activity is most widely used to determine in vitro anti-obesity activity since it is one of the important enzymes in hydrolyzing triglyceride and thus absorbing lipids in the small intestine [28]. The synergistic effect of GSF and HLAB in the current study could be due to the activation of multifunctional pathways by a combination of bioactive components (cellular components in HLAB and polyphenols in GSF), as well as the alteration of intestinal microbiota. Further study is required to determine the role of intestinal microbiota in the synergistic anti-obesity action of HLAB and GSF. These studies suggest that kefir HLAB are functional ingredients that can be used to alleviate obesity, especially in combination with GSF.

In summary, the combination of wine GSF and paraprobiotic kefir HLAB synergistically prevented the increase of HFFrD-induced body weight, adipose tissue weight, and plasma triglyceride concentrations. Cellular EPS extracted kefir LAB can lower lipid accumulation in 3T3-L1 cells, indicating that it can be a potential bioactive component to exert the anti-obesity effect of HLAB. The anti-obesity effect was in part mediated via a decrease in the expression of adipocyte genes related to fatty acid synthesis and inflammation, which was significantly correlated with obesity physiological biomarkers (body weight gain and adipose tissue weight).

## 5. Conclusions

This study suggests that a combination of prebiotic Chardonnay GSF and paraprobiotic kefir HLAB are potential functional food ingredients that synergistically alleviate obesity, especially for immunocompromised individuals. This combination acts partly via alterations in adipogenesis and adipocyte inflammation.

## Figures and Tables

**Table 1:** Animal diet composition.

Ingredient	CON	2.5% GSF
Lard fat	225	225
Soybean oil	27	25
Cholesterol	0.8	0.8
MCC ^a^	50	35.7
GSF ^b^	0	25
Casein	200	200
Corn starch	91.2	82.5
Fructose	355	355
l-cystine	3	3
Choline bitartrate	3	3
Mineral mix	35	35
Vitamin mix	10	10
Total diet (g)	1000	1000
Fat %	46.7	46.8
Protein %	16.7	16.8
Carbohydrate %	36.6	36.4

^a^ MCC, microcrystalline cellulose; ^b^ GSF, Chardonnay grape seed flour.

**Table 2:** PCR primer sequences used in gene expression analysis.

Gene	Forward (5′-3′)	Reverse (5′-3′)
*Aox*	GTTGATCACGCACATCTTGG	TGGCTTCGAGTGAGGAAGTT
*Cd36* ^1^	CCGGGCCACGTAGAAAACA	CCTCCAAACACAGCCAGGAC
*Fasn* ^2^	AGCACTGCCTTCGGTTCAGTC	AAGAGCTGTGGAGGCCACTTG
*Hp* ^3^	CGAGAAGAAAAACTTGACGA	TCACGTACACACCATACTCAG
*Wfdc21*	TGAGACCTCTGCAGCTTTTAG	ACAGATGTGACTGCATCCAATA
*36B4* ^4^	TCTAGGACCCGAGAAGACCTC	GTTGTCAAACACCTGCTGGAT

^1^: [15]; ^2^: [16]; ^3^: [17]; ^4^: [18].

**Table 3:** Metabolic parameters in HFFr diet-induced obese (DIO) mice fed 5% microcrystalline cellulose (CON, control), 2.5% wine grape seed flour (GSF), 2.5% GSF with heat-treated LAB (GSF+HLAB), and heat-treated LAB (HLAB) for 8 weeks.

	CON	GSF	GSF+HLAB	HLAB
**Total cholesterol (mg/dL)**	136 ± 8.8	129 ± 5.7	118 ± 4.7	129 ± 8.1
**HDL cholesterol (mg/dL)**	79 ± 2.9	74 ± 3.6	68 ± 6.4	78 ± 5.4
**LDL cholesterol (mg/dL)**	17 ± 0.7	16 ± 1.0	18 ± 2.4	17 ± 1.0
**Triglycerides (mg/dL)**	99 ± 8.9 ^a^	78 ± 5.7 ^a^	72 ± 4.7 ^b^	88 ± 8.1 ^a^
**Glucose (mg/dL)**	203 ± 18.6	189 ± 18.2	209 ± 21.6	209 ± 25.8

Data are means ± SEM, values not sharing a common letter differed significantly at *p* < 0.05.

**Table 4:** Correlation between the adipocyte mRNA expression and physiological parameters in HFFrD-IO mice fed 5% microcrystalline cellulose (MCC, CON), 2.5% wine grape seed flour (GSF), 2.5% GSF with heat-treated LAB (GSF+HLAB), and heat-treated LAB (HLAB) for 8 weeks.

	Bodyweight Gain	Adipose Tissue Weight	Triglycerides
***Fabp4***	0.57 *	0.66 ^#^	0.38
***Fasn***	0.46 *	0.50 *	0.23
***Wfdc21***	0.66 ^#^	0.61 ^#^	0.40
***Hp***	0.56 *	0.79 ^#^	0.57 *

* *p* < 0.05; ^#^ *p* < 0.01.
